# Evaluation of the Effect of Intravenous Dexamethasone on the Duration of Spinal Anaesthesia in Parturients Undergoing Lower Segment Caesarean Section

**DOI:** 10.7759/cureus.37549

**Published:** 2023-04-13

**Authors:** Manisha Manohar, Suresh Singhal, Nitika Goyal

**Affiliations:** 1 Anesthesiology, Pandit Bhagwat Dayal Sharma Post Graduate Institute of Medical Sciences, Rohtak, IND; 2 Anasthesiology, Pandit Bhagwat Dayal Sharma Post Graduate Institute of Medical Sciences, Rohtak, IND

**Keywords:** dexamethasone, bupivacaine, spinal, anesthesia, lscs, neuraxial, intrathecal, additives, motor block, sensory block

## Abstract

Introduction

Dexamethasone is shown to prolong the duration of nerve blocks when administered perineurally as well as intravenously. The effect of intravenous dexamethasone on the duration of hyperbaric bupivacaine spinal anesthesia is lesser known. We conducted a randomized control trial to determine the effect of intravenous dexamethasone on the duration of spinal anesthesia in parturients undergoing lower-segment cesarean section (LSCS).

Methods

Eighty parturients planned for LSCS under spinal anesthesia were randomly allocated to two groups. Patients in group A were administered dexamethasone intravenously, and group B received normal saline intravenously before spinal anesthesia. The primary objective was to determine the effect of intravenous dexamethasone on the duration of sensory and motor block after spinal anesthesia. The secondary objective was to determine the duration of analgesia and complications in both groups.

Result

The total duration of the sensory and motor blocks in group A was 118.38 ± 19.88 minutes and 95.63 ± 19.91 minutes, respectively. The entire sensory and motor blockade duration in group B was 116.88 ± 13.48 minutes and 97.63 ± 15.15 minutes, respectively. The difference between the groups was found to be statistically insignificant.

Conclusion

Intravenous 8 mg dexamethasone in patients planned for LSCS under hyperbaric spinal anesthesia does not prolong the sensory or motor block duration compared to placebo.

## Introduction

Spinal anesthesia is the technique of choice for cesarean delivery because it avoids the risks of general anesthetics and provides effective pain control, early ambulation, and fast return to daily activities, thereby increasing the quality of life [1]. Intrathecal block, however, has the disadvantage of limited duration sensory block.

Dexamethasone is an effective adjuvant for prolonging peripheral nerve block duration with minimal side effects. Block can be prolonged by perineural [2-4] or intravenous administration of dexamethasone [5,6]. Even though perineural administration of dexamethasone seems to be more effective than systemic use, and many providers use systemic dexamethasone to avoid mixing drugs that were not designed to be administered together, circumvent the problem of off-label perineural use, and profit from antiemetic effects of systemic dexamethasone [7]. Doses between 4 to 10 mg have been used in adults [8,9]. The precise mechanism of action is not understood [7], and the potential for neurotoxic side effects is not adequately studied. However, the postulated mechanism for prolonging the duration of peripheral nerve blocks is attributed to the attenuation of the release of inflammatory mediators and inhibition of transmission in thin unmyelinated C fibers [10,11].

Intrathecal administration of dexamethasone prolongs the duration of sensory block [12,13]. However, the effect of intravenous dexamethasone on the duration of spinal anesthesia has been assessed by a few studies with conflicting results [14,15]. We hypothesized that preoperative administration of 8 mg intravenous dexamethasone would significantly prolong the duration of spinal anesthesia and conducted a randomized control trial with the primary objective of determining the effect of intravenous dexamethasone on the sensory and motor block duration after spinal anesthesia. The secondary objective was to determine the duration of analgesia and complications.

## Materials and methods

This prospective, randomized, double-blind, placebo-controlled study was conducted after approval from the biomedical research ethics committee, Pandit Bhagwat Dayal Sharma Post Graduate Institute of Medical Sciences (PGIMS), Rohtak (Number: BREC/Th/20/Anaesth../029). The trial was registered with the Clinical Trial Registry - India under the number CTRI/2022/04/041979. Parturients between 18 to 35 years of age belonging to the American Society of Anesthesiologists (ASA) physical status II undergoing lower segment cesarean section (LSCS) under spinal anesthesia were included in the study. Written informed consent was taken from the patient before enrolling in the study. Patients who refused to give consent, patients with pregnancy-induced hypertension, pre-existing hemodynamic instability, diabetes, cardiac ailment, pre-existing neuropathy, preoperative use of systemic corticosteroids, history of allergy or hypersensitivity to local anesthetics, dexamethasone or other drugs used in this study, any contraindication to spinal anesthesia (coagulopathy, local infection at the site of injection), were excluded from the study.

Our estimated sample size was calculated with reference to a previous study by Shalu et al., where the mean duration of sensory block was 162.50 min in group D and 106.17 min in group S [14]. A minimum sample size of 37 patients per group with an effect size of 0.75 provided a 90% power for detecting a significant difference between the two groups at an alpha level of 0.05. We included 40 patients in each group.

During the pre-anesthesia checkup, clinical history followed by general physical and systemic examination was done. Routine investigations like hemoglobin, bleeding time, clotting time, and urine examination were carried out in all the patients. Any other relevant investigations were done as and when required. Informed written consent to participate in the study was taken from all the patients. The patients were fasting for six hours for solids and two hours for liquids before surgery. Aspiration prophylaxis was administered to all patients as per the standard protocol of the institute. After arrival in the operating room, ASA standard monitors were placed. Baseline vital parameters were recorded. An intravenous line (IV) was secured with an 18 G cannula, and an infusion of Ringer's lactate was started. Patients were randomly allocated by sealed, opaque envelopes into one of the two groups according to a computer-generated sequence of random numbers.

Patients in group A, the dexamethasone group, (n=40) received 8 mg (2 ml dexamethasone + 8 ml NS= 10 ml total) of dexamethasone intravenously over 5-10 min. Patients in group B, the placebo group, (n=40) received 10 ml of normal saline intravenously over 5-10 minutes.

Drugs used in the study were prepared by an investigator not involved with patient enrollment or data collection. Before positioning the patient for spinal anesthesia, dexamethasone/ normal saline was administered as per the allocated group. Under all aseptic precautions, 2ml of hyperbaric bupivacaine 0.5% was administered intrathecally using a 25 G Quincke's spinal needle at L4-L5 or L3-L4 intervertebral space. At the end of the intrathecal injection, the patient was positioned supine for surgery. Sensory blockade was evaluated with a blunt 22 G hypodermic needle, while motor blockade was assessed using the Bromage score [16]. The level of the sensory and motor block was assessed by a blinded investigator every two minutes for the first 10 minutes, then every five minutes for the next 20 minutes, followed by 15 min intervals till the regression of sensory block to L1 and restoration of motor power to Bromage score II. After completion of the surgery, patients were continuously monitored in the post-operative area till the predefined end-points for regression of sensory and motor block were attained. At the time of the first complaint of pain by the patient, rescue analgesia was administered with Paracetamol 1gm IV.

Statistical analysis

Statistical analysis was performed using Statistical Package for Social Sciences (SPSS) program for Windows, version 17.0 (IBM Inc., Armonk, New York). Continuous variables were presented as mean ± SD, and categorical variables were presented as absolute numbers and percentages. Data were checked for normality before statistical analysis. Normally distributed continuous variables were compared using the unpaired T-test, whereas the Mann-Whitney U test was used for those variables that were not normally distributed. Categorical variables were analyzed using the Pearson Chi-square or Fisher's exact tests. A p-value of <0.05 was considered statistically significant

## Results

The mean age, height, weight, and hemodynamic parameters were comparable in both groups (Tables 1, 2).

**Table 1:** Patient characteristics

	Group A (dexamethasone group) n=40 (mean ± SD)	Group B (placebo group) n=40 (mean ± SD)	p-value
Age (years)	25.30 ± 4.09	25.23 ± 3.13	0.927
Height (cm)	158.30 ± 6.95	158.85 ± 6.43	0.714
Weight (kg)	59.68 ± 4.93	57.20 ± 4.57	0.833

**Table 2:** Baseline hemodynamic parameters

Baseline hemodynamic parameters	Group A (dexamethasone group) n=40 (mean ± SD)	Group B (placebo group) n=40 (mean ± SD)	p-value
Heart rate (beats/min)	90.03 ± 10.64	93.88 ± 10.40	0.106
Systolic blood pressure (mmHg)	119.75 ± 8.66	119.73 ± 8.38	0.990
Diastolic blood pressure (mmHg)	73.85 ± 8.04	71.48 ± 6.90	0.160
Mean blood pressure (mmHg)	89.08 ± 7.76	87.38 ± 7.04	0.308

The time between intrathecal injection of local anesthetic and T6 or higher dermatome blockade determined by 22G blunt hypodermic needle tip was recorded as the onset of sensory blockade. The mean onset of sensory block in group A was 5.85 ± 0.70 minutes, while the mean onset of sensory blockade in group B was 5.73 ± 0.96 minutes (Table 3). The difference between the two groups was statistically insignificant (p=0.508). The peak level of sensory blockade attained was defined as the maximum level achieved at 20 minutes from intrathecal injection of local anesthetic. Ten patients attained a peak level of T4 in group A and six patients in group B. Three patients in group A attained a peak level of T5. In comparison, only two patients achieved a peak level of T5 in group B. Twenty-six patients in group A attained a peak level of T6, while 31 patients attained a peak level of T6 in group B. A peak level of T8 was attained by only one patient in both groups (Table 3). The two groups were found to be statistically comparable (p=0.651). The duration of sensory blockade was defined as the time of regression of sensory blockade from peak sensory level attained at 20 minutes to L1 dermatome. The total duration of the sensory block was 118.38 ± 19.88 minutes in group A and 116.88 ± 13.48 minutes in group B (Table 3) (p=0.694).

**Table 3:** Sensory block characteristics in two groups

	Group A (dexamethasone group) n=40 (mean ± SD)	Group B (placebo group) n=40 (mean ± SD)	p-value
Onset of sensory blockade (min)	5.85±0.70	5.73±0.96	0.508
Peak sensory level attained			
T4	10	6	0.651
T5	3	2	
T6	26	31	
T8	1	1	
Duration of sensory block (min)	118.38±19.88	116.88±13.48	0.694

The time between intrathecal injection of the local anesthetic to Bromage score of three or higher was recorded as the onset of motor blockade. The mean onset of the motor blockade in group A was 7.33 ± 1.02 minutes, and the mean onset in group B was 7.25 ± 1.08 minutes (Table 4). The difference between the two groups was statistically insignificant (p=0.751). The peak level of motor blockade attained was defined as the maximum degree of motor blockade achieved at 20 minutes after the intrathecal injection of local anesthetic. The peak level of motor blockade attained was grade four in both groups (Table 4). The duration of motor blockade was defined as the time of regression of motor block from peak motor blockade at 20 minutes to Bromage score of two. The total duration of motor blockade was 95.63 ± 19.91 minutes in group A and 97.63 ± 15.15 minutes in group B (Table 4). The two groups were statistically comparable (p=0.615).

**Table 4:** Motor block characteristics in two groups

	Group A (dexamethasone group) n=40 (mean ± SD)	Group B (placebo group) n=40 (mean ± SD)	p-value
Onset of motor blockade (min)	7.33 ± 1.02	7.25 ± 1.08	0.751
Peak level of motor block attained	Grade 4 in all patients	Grade 4 in all patients	NA
Duration of motor blockade (min)	95.63±19.91	97.63±15.15	0.615

The duration of postoperative analgesia was defined as the time from completion of surgery to the time of the first complaint of pain by the patient. The total duration of postoperative analgesia was 34.68 ± 12.25 minutes in group A and 32.28 ± 14.87 minutes in group B (Table 5). The two groups were statistically comparable (p=0.433). The total duration of analgesia was defined as the time from the intrathecal injection to the first complaint of pain by the patient. The entire duration of analgesia was 105.38 ± 19.60 minutes in group A and 104.85 ± 12.18 minutes in group B (Table 5). There was no statistically significant difference between the two groups (p=0.886)

**Table 5:** Duration of analgesia and complications in two groups

	Group A (dexamethasone group) n=40 (mean ± SD)	Group B (placebo group) n=40 (mean ± SD)	p-value
Duration of post- operative analgesia (min)	34.68±12.25	32.28±14.87	0.433
Total duration of analgesia (min)	105.38±19.60	104.85±12.18	0.886
Number of episodes of bradycardia	1	0	0.314
Number of episodes of hypotension	9	8	0.728

Bradycardia was defined as a heart rate <20% of the baseline heart rate. Only one patient had an episode of bradycardia in group A, while no patient had bradycardia in group B. The difference between the two groups was statistically insignificant (p=0.314) (Table 5). Hypotension was defined as blood pressure <20% of baseline blood pressure. Nine patients out of 40 in group A and eight out of 40 in group B had episodes of hypotension. The two groups were found to be statistically comparable (p=0.728) (Table 5).

## Discussion

This trial demonstrates that preoperative administration of intravenous dexamethasone does not prolong the duration of spinal anesthesia after intravenous dexamethasone administration in patients planned for cesarean section under spinal anesthesia. The findings of our study are concordant with the results reported by Guay et al., who demonstrated that the time to complete regression of sensory blockade was 356 minutes in patients who received intravenous dexamethasone before isobaric spinal anesthesia and 344 minutes in the placebo group (p=0.403) [15]. Contrary to our study, Shalu et al. found that the mean duration of sensory block is significantly prolonged in patients who received intravenous dexamethasone before spinal anesthesia [14]. The authors reported that the mean duration of sensory block was significantly higher in the dexamethasone group, i.e., 162.50 minutes, as compared to the placebo group, i.e., 106.17 minutes (p<0.001), with a mean difference of 64 minutes between the control and the interventional group in contrast to a difference of two minutes demonstrated in our study (p=0.694). However, these authors have not mentioned the criteria for determining the duration of the sensory block. They also administered ondansetron before intrathecal anesthesia with 10 mg (2 ml) of 0.5% hyperbaric bupivacaine. The anti-nociceptive effect of 5-HT3 antagonist has been postulated to be produced by an action on the neurons in the spinal cord that codes and transmits peripheral nociceptive stimuli [17]. This could be the probable reason for the difference in our findings. Parthasarathy et al. also found that in patients who received dexamethasone before spinal anesthesia, the time of regression of the sensory block to L1 increased by 34 minutes [18]. They reported that the mean time required for the complete reversal of sensory block was 254.67 ± 24.09 minutes in the dexamethasone group and 220.17 ± 24.93 minutes in the placebo group, which was found to be statistically significant (p<0.001). A higher dose (15 mg) of hyperbaric bupivacaine was used by Parthasarathay et al., contrary to the 10 mg of bupivacaine in our study translates into the prolonged duration of sensory block observed by them [18].

We found that the duration of motor block is not altered with preoperative administration of intravenous dexamethasone. Shalu et al. also observed a statistically insignificant difference in the mean duration of motor block in both groups (169.5 minutes in the dexamethasone group vs. 163.16 minutes in the control group) [14]. Guay et al. also found that patients who received dexamethasone intravenously regressed to a Bromage score of zero after 298 minutes, while those who received a placebo took 274 minutes to revert to a Bromage score of zero (p=0.101) [15]. Contrary to our results, Parthasarthay et al. found that the regression of motor block to Bromage score zero takes 220.17±24.93 minutes in patients who receive dexamethasone while the motor block regresses 17 minutes early (203.83±24.09 minutes) in patients not administered dexamethasone [18]. The authors found a statistically significant difference in the duration of the motor block between the intervention and control groups in their study (p<0.001).

Studies have confirmed the analgesic effect of dexamethasone after different surgeries [19,20]. Some studies suggest a delayed analgesic effect [21], while some reveal the rapid onset of pain reduction [22]. Inhibition of prostaglandin, bradykinin production, reduction of tissue swelling, and inhibition of nerve compression by inflammatory tissue are the suggested mechanism for analgesic effect. The duration of postoperative analgesia, however, was similar in both groups in our study. Our findings concord with the results illustrated in the study by Guay et al., where the authors found no significant effect of intravenous dexamethasone on the time to first rescue analgesia and 24-hour opioid consumption after spinal anesthesia with isobaric bupivacaine [15]. Parthasarathy et al. reported that intravenous dexamethasone, after spinal anesthesia, efficiently reduces postoperative pain [18]. They recorded the duration of postoperative analgesia as the time from the completion of surgery to the time of the first complaint of pain by the patient. They reported that the patients who received dexamethasone requested first rescue analgesia after 297.83 ± 29.56 minutes, while those who received a placebo asked much earlier, i.e., 175.50 ± 29.17 minutes (p<0.001). Contrary to this, we found that the obstetric patients who received dexamethasone reported pain after 34.68 ± 12.25 minutes of completion of the surgery, while those who were administered normal saline requested analgesia after 32.28 ± 14.87 minutes (p=0.433). The total duration of postoperative analgesia was similar in both groups in our study. This difference is probably because Parthasarathy et al. used 15 mg of bupivacaine which is 5 mg higher than the dose used in our research [18]. In our study, we took a homogenous cohort of obstetric patients, while Parthasarathy et al. conducted the study on non-obstetric patients planned for surgery under spinal anesthesia. Shalu et al. reported that the mean time to the requirement of first rescue analgesia was 8.67 hours in patients who received dexamethasone and 4.40 hours in those who did not [14]. Ondansetron has been postulated to have an anti-nociceptive effect through its effect on the neurons in the spinal cord that code and transmit peripheral nociceptive stimuli [21]. The administration of intravenous ondansetron before intrathecal anesthesia could have prolonged the sensory block duration in the study by Shalu et al. [14]

The leading cause of post-spinal hypotension is the decrease in the sympathetic outflow, causing arterial vasodilation and a reduction of venous return and, consequently, the activation of the Bezold Jarish reflex that elicits the triad of bradycardia, vasodilation, and further hypotension. In concordance with our study, Parthasarthay et al. also reported that preoperative administration of dexamethasone does not alter the incidence of hypotension [18]. They reported that four out of 30 patients (13.3%) in the dexamethasone group and seven out of 30 patients (23.3%) in the normal saline group developed hypotension after intrathecal anesthesia (p=0.317). Guay et al. also reported a statistically insignificant difference in the episode of hypotension when compared between the dexamethasone and placebo groups [15]. They found that 17% of patients (five out of 30) who received intravenous dexamethasone had episodes of hypotension, while only 10% of patients (three out of 30) who received a placebo had a bout of hypotension (p=0.712).

Though statistically insignificant, our study also shows that the episodes of hypotension were more in the dexamethasone group. Ashoor et al. demonstrated a favorable response regarding the efficacy of a single preoperative dose of dexamethasone 8 mg intravenous infusion to attenuate post-spinal anesthesia hypotension in geriatric patients [23]. The authors reported that 14.5% of the patients (eight out of 55) in the dexamethasone group developed hypotension as compared to 32.5% of patients (18 out of 55) in the placebo group (p=0.025). They reported that the need for ephedrine is significantly lower in the dexamethasone group. This difference in the results could be attributed to the different patient cohorts used in our study and the difference in the timing of the administration of dexamethasone. Guay et al., in their study on patients undergoing orthopedic, urological, or other surgeries under spinal anesthesia, also reported that the incidence of bradycardia was similar (17%) in both the dexamethasone and placebo groups (p=1) [15]. The authors reported that 17% of patients (five out of 30) who received intravenous dexamethasone had episodes of hypotension, while only 10% of patients who received a placebo had a bout of hypotension (p=0.712). The authors concluded that there was no significant effect of intravenous dexamethasone on the incidence of hypotension as well as bradycardia.

Ashoor et al. reported that seven out of 55 patients (17.5%) had an episode of bradycardia in the dexamethasone group, and 11 out of 55 patients (20%) in the control group had an episode of bradycardia with p=0.303 [23]. Hence they concluded that intravenous dexamethasone has no significant effect on intraoperative episodes of bradycardia after spinal anesthesia. However, Parthasarathy et al. reported relatively higher values of intraoperative heart rate at various intervals during surgery among patients who received intravenous dexamethasone compared to those who did not. They found this difference to be highly statistically significant (p=0.071) [17].

The strengths of our study include appropriately matched control and interventional groups in terms of demographic parameters, baseline hemodynamic parameters, and duration of surgery. All sensory and motor blockade parameters assessed by anaesthesiologists were defined in terms of objective endpoints to ensure the appropriate data collection. The drawback of our trial was that different anesthesiologists administered the spinal anesthesia, and there was no objective assessment of the speed of injecting the intrathecal drug. Patients were monitored only until the first complaint of pain and rescue analgesia administration; hence, dexamethasone's effect on long-term postoperative pain was not determined.

## Conclusions

We conclude that administering intravenous 8 mg dexamethasone in patients planned for LSCS under spinal anesthesia does not prolong the sensory or motor block duration compared to placebo. Studies with larger sample sizes, focusing on nonobstetric patient populations, would be of clinical interest to determine further whether intravenous dexamethasone has any effect on spinal anesthesia or not.
